# Circulating β cell‐specific CD8^+^ T cells restricted by high‐risk HLA class I molecules show antigen experience in children with and at risk of type 1 diabetes

**DOI:** 10.1111/cei.13391

**Published:** 2019-11-10

**Authors:** L. Yeo, I. Pujol‐Autonell, R. Baptista, M. Eichmann, D. Kronenberg‐Versteeg, S. Heck, G. Dolton, A. K. Sewell, T. Härkönen, M.‐L. Mikk, J. Toppari, R. Veijola, M. Knip, J. Ilonen, M. Peakman

**Affiliations:** ^1^ Department of Immunobiology Faculty of Life Sciences and Medicine King’s College London London UK; ^2^ National Institute of Health Research Biomedical Research Centre at Guy’s and St Thomas’ Hospital and King’s College London London UK; ^3^ Division of Infection and Immunity School of Medicine and Systems Immunity Research Institute Cardiff University Cardiff UK; ^4^ Research Program for Clinical and Molecular Metabolism Faculty of Medicine University of Helsinki Helsinki Finland; ^5^ Immunogenetics Laboratory Institute of Biomedicine University of Turku Turku Finland; ^6^ Department of Paediatrics University of Turku and Turku University Hospital Turku Finland; ^7^ Institute of Biomedicine Research Centre for Integrative Physiology and Pharmacology University of Turku Turku Finland; ^8^ Department of Paediatrics PEDEGO Research Unit Medical Research Centre Oulu University Hospital and University of Oulu Oulu Finland; ^9^ Children’s Hospital University of Helsinki and Helsinki University Hospital Helsinki Finland; ^10^ Department of Pediatrics Tampere University Hospital Tampere Finland; ^11^ Folkhälsan Research Centre Helsinki Finland; ^12^ Clinical Microbiology Turku University Hospital Turku Finland; ^13^ King’s Health Partners Institute of Diabetes, Endocrinology and Obesity London UK

**Keywords:** CD8^+^ T cells, HLA‐B*39, HLA‐A*24, type 1 diabetes

## Abstract

In type 1 diabetes (T1D), autoreactive cytotoxic CD8^+^ T cells are implicated in the destruction of insulin‐producing β cells. The HLA‐B*3906 and HLA‐A*2402 class I genes confer increased risk and promote early disease onset, suggesting that CD8^+^ T cells that recognize peptides presented by these class I molecules on pancreatic β cells play a pivotal role in the autoimmune response. We examined the frequency and phenotype of circulating preproinsulin (PPI)‐specific and insulin B (InsB)‐specific CD8^+^ T cells in HLA‐B*3906 ^+^ children newly diagnosed with T1D and in high‐risk HLA‐A*2402 ^+^ children before the appearance of disease‐specific autoantibodies and before diagnosis of T1D. Antigen‐specific CD8^+^ T cells were detected using human leucocyte antigen (HLA) class I tetramers and flow cytometry was used to assess memory status. In HLA‐B*3906 ^+^ children with T1D, we observed an increase in PPI_5–12_‐specific transitional memory CD8^+^ T cells compared to non‐diabetic, age‐ and HLA‐matched subjects. Furthermore, PPI_5–12_‐specific CD8^+^ T cells in HLA‐B*3906 ^+^ children with T1D showed a significantly more antigen‐experienced phenotype compared to polyclonal CD8^+^ T cells. In longitudinal samples from high‐risk HLA‐A*2402 ^+^ children, the percentage of terminal effector cells within the InsB_15–24_‐specific CD8^+^ T cells was increased before diagnosis relative to samples taken before the appearance of autoantibodies. This is the first study, to our knowledge, to report HLA‐B*3906‐restricted autoreactive CD8^+^ T cells in T1D. Collectively, our results provide evidence that β cell‐reactive CD8^+^ T cells restricted by disease‐associated HLA class I molecules display an antigen‐experienced phenotype and acquire enhanced effector function during the period leading to clinical diagnosis, implicating these cells in driving disease.

## Graphical abstract

In this study, we characterised circulating autoreactive CD8^+^ T cells in children with type 1 diabetes with HLA-B*3906^+^ and HLA-A*2402^+^ genotypes which are associated with increased risk and early onset of disease. In HLA-B*3906^+^ children with newly diagnosed type 1 diabetes, we observed an increase in PPI_5–12_‐specific transitional memory CD8^+^ T cells compared to non‐diabetic, age‐ and HLA‐matched subjects. Furthermore, PPI_5–12_‐specific CD8^+^ T cells in HLA‐B*3906^+^ children with T1D showed a significantly more antigen‐experienced phenotype compared to polyclonal CD8^+^ T cells. Further, in longitudinal samples from high-risk HLA-A*2402^+^ children, the percentage of terminal effector cells within the InsB_15–24_‐specific CD8^+^ T cell population was increased before diagnosis relative to samples taken before the appearance of autoantibodies.

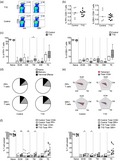

## Introduction

Type 1 diabetes is an autoimmune disease in which insulin‐secreting β cells of the pancreatic islets are selectively destroyed 1, 2. Several lines of evidence point to CD8^+^ cytotoxic T cells as critical players in β cell destruction. Firstly, CD8^+^ T cells are the dominant cell type found in insulitic lesions in the pancreas of patients with type 1 diabetes 3, 4, 5, and hyperexpression of human leucocyte antigen (HLA) class I molecules in this lesion has the potential to enhance peptide presentation to infiltrating CD8^+^ T cells 3, 4, 5, 6, 7, 8. Secondly, CD8^+^ T cells with specificity for β cell antigens are present in the blood and islets of individuals with type 1 diabetes 9, 10, 11, 12, 13, 14. Thirdly, CD8^+^ T cell clones recognizing β cell epitopes are able to kill isolated β cells *in vitro*, exemplifying the potential for CD8^+^ T cells to constitute major effectors of β cell death 13, 15. Lastly, recent success in delaying β cell loss using immunotherapy targeted at effector CD8^+^ T cells suggests that CD8^+^ T cells are a dominant killing pathway 16.

These immunological observations associate the CD8^+^ T cell pathway with disease, a proposal that is further supported by genetic studies. These highlight specific HLA class I loci with type 1 diabetes predisposition independently of linkage disequilibrium with HLA class II 17. For example, HLA‐B*39 was identified as a major genetic risk locus in a large‐scale study of single nucleotide polymorphisms associated with allelic forms of HLA class I genes 17. HLA‐B*39 is relatively rare, being present in 0·5–1·2% of European, North American and Australian populations and 0·1% of Southeast Asian populations 18, 19. HLA‐B*39 polymorphism is associated with increased susceptibility to type 1 diabetes, providing an odds ratio of 2·41 in a case–control set, and a relative risk of 3·55 in affected sibling‐pair families 17. In addition, HLA‐B*39 polymorphism associates with a lower age of type 1 diabetes diagnosis, and the HLA‐B*3906 subtype is linked to a lower age of diagnosis by an average of 1·7–3·7 years in several independent studies 17, 20, 21, 22, 23, 24. HLA‐B*39 polymorphism is also associated with accelerated disease progression in children from the point of autoantibody development to clinical diagnosis, implying more rapid β cell destruction 25, 26. Furthermore, the HLA‐B*3906 variant significantly enhances the risk of type 1 diabetes in individuals carrying specific *HLA‐DR/DQ* haplotypes; namely, DRB01*0404‐DQB1*0302 21, 23, 26, 27, 28, 29 and DRB1*08–DQB1*0402 24, 29, 30, 31.

At the *HLA‐A* locus, the HLA‐A*24 allele has a strong type 1 diabetes disease‐predisposing effect. The HLA‐A*24 supertype is present in 12–20% of Caucasian and 60% of Japanese populations, with HLA‐A*2402 being the most common variant 32, 33. Polymorphisms associated with the HLA‐A*24 allele confer a higher disease risk, with an odds ratio of 1·5 17, and share disease‐influencing features in common with HLA‐B*39. For example, HLA‐A*24 is significantly associated with a younger age at diagnosis 17, 34, 35, 36. HLA‐A*24 polymorphisms are an independent predictor of progression to type 1 diabetes in autoantibody‐positive first‐degree relatives of individuals with type 1 diabetes 37 and are associated with accelerated disease progression from seroconversion to clinical diagnosis 26, 37, 38, 39. Furthermore, the presence of HLA‐A*24 has been associated with early and complete β cell destruction after diagnosis 40, 41, and with poor functional outcome in islet transplant recipients 42.

Collectively, these studies prompt questions in relation to the presentation of β cell autoantigens to CD8^+^ T cells by HLA‐B*39‐ and HLA‐A*24‐encoded HLA class I molecules and the potential of these events to drive β cell destruction and accelerate type 1 diabetes progression. We recently identified preproinsulin epitopes naturally processed and presented by HLA class I molecules encoded by HLA‐B*3906 and HLA‐A*2402 and associated with disease risk and progression 43, 44. In the present study, we used this insight to examine the repertoires of HLA‐B*3906‐ and HLA‐A*2402‐restricted β cell‐specific CD8^+^ T cells in children with, or at risk of, type 1 diabetes. We analysed samples from Finnish cohorts in which the independent and additive effects of these high‐risk alleles on type 1 diabetes risk are well documented 21, 23, 26, 27, 29. We provide evidence that autoreactive CD8^+^ T cells restricted by HLA‐B*3906 and HLA‐A*2402‐encoded HLA class I molecules display an antigen‐experienced phenotype and acquire enhanced effector function during the period leading to clinical diagnosis of type 1 diabetes.

## Materials and methods

### Subjects

The HLA‐B*3906 ^+^ study cohort comprised 10 children with newly diagnosed type 1 diabetes (time after clinical diagnosis 2–10 days; median age 2·7 years) and seven non‐diabetic children recruited as control subjects, with a median age of 2·2 years (Table 1). Additionally, one child had blood drawn at the age of 3·3 years, 5 years before  she was diagnosed with  type 1 diabetes at the age of 8·6 years. All children with newly diagnosed type 1 diabetes were recruited from the Finnish Pediatric Diabetes Register 45. The Finnish Pediatric Diabetes Register study was approved by the Ethical Committee of the Hospital District of Helsinki and Uusimaa and the register steering committee. The parents of the children gave their written informed consent. Five control subjects were healthy siblings of type 1 diabetes‐affected children recruited from the Finnish Pediatric Diabetes Register, and two control subjects had HLA class II genotypes associated with increased risk for type 1 diabetes and were recruited as participants in the Finnish Type 1 Diabetes Prediction and Prevention (DIPP) study 46, 47. Six of seven control subjects were autoantibody‐negative at the time of blood draw. Although one of the seven control subjects was positive for insulin autoantibodies (IAA) this subject was included, as it known that the number of detectable autoantibodies is related to risk of progression to type 1 diabetes, with seropositivity for two or more islet autoantibodies conferring high risk for developing type 1 diabetes, whereas positivity for a single autoantibody may represent non‐progressive or regressive β cell autoimmunity 48. The DIPP study was approved by local Ethics Committees and all families participating in the study provided written informed consent.

**Table 1:** HLA‐B*3906 ^+^ subject information

	T1D subjects	Controls
*n*	10	7
Sex *n* (%)		
Male	6 (60%)	3 (43%)
Female	4 (40%)	4 (57%)
Age, median (IQR) (years)	2·7 (1·8–3·3)	2·2 (1·8–4·1)
Age at diagnosis, median (IQR) (years)	2·6 (1·8–3·3)	n.a.
T1D duration (days)	5 (3–7)	n.a.
Autoantibody‐positive, *n* (%)	9 (90%)	1 (14%)

T1D = type 1 diabetes; IQR = interquartile range; n.a. = not available.

The HLA‐A*2402 ^+^ study cohort comprised 15 children sampled before diagnosis of type 1 diabetes (median time before clinical diagnosis = 7 months; median age = 5·0 years) and 15 autoantibody‐negative non‐diabetic children recruited as control subjects, with a median age of 4·9 years (Table 2). Of the 15 children who provided samples before diagnosis of type 1 diabetes, 11 had blood drawn before seroconversion to autoantibody positivity (median time before seroconversion = 5 months; median age = 1·5 years) (Table 3). All HLA‐A*2402 ^+^ study subjects participated in the DIPP study with regular follow‐up and had HLA class II genotypes associated with increased risk for type 1 diabetes 49. Positivity for islet autoantibodies was analysed in all subjects participating in either the Finnish Pediatric Diabetes Register or the DIPP study at the time of sampling, as previously described 46, 50. Positivity for Epstein–Barr virus (EBV) immunoglobulin (Ig)G antibodies was analysed in 12 of 15 subjects whose samples were stained with EBV tetramers. Serum samples were tested for anti‐viral capsid antigen (VCA) and anti‐early antigen (EA) IgG using an automated immunoanalyzer based on enzyme‐linked fluorescence (ELFA) technology (VIDAS^®^; bioMérieux, Marcy l’Etoile, France). Results were reported as negative or positive with cut‐offs of 0·1 and 0·23 test value.

**Table 2:** HLA‐A*2402 ^+^ subject information

	T1D subjects	Controls
*n*	15	15
Sex *n* (%)		
Male	10 (66%)	10 (66%)
Female	5 (33%)	5 (33%)
Age, median (IQR) (years)	5·0 (1·7–9·0)	4·9 (1·8–8·5)
Age at diagnosis, median (IQR) (years)	5·9 (3·5–9·3)	n.a.
Time before diagnosis, median (IQR) (months)	7 (4–21)	n.a.
Autoantibody‐positive, *n (*%)	14 (93%)	0 (0%)

T1D = type 1 diabetes; IQR = interquartile range; n.a. = not available.

**Table 3:** Pre‐ and post‐seroconversion HLA‐A*2402 ^+^ subject information

	T1D subjects pre‐seroconversion	T1D subjects post‐seroconversion
*n*	11	11
Sex *n* (%)		
Male	9 (82%)	9 (82%)
Female	2 (18%)	2 (18%)
Age, median (IQR) (years)	1·5 (1·0–2·1)	5·8 (3·4–9·8)
Age at seroconversion, median (IQR) (years)	2·5 (1·3–3·2)	2·5 (1·3–3·2)
Age at diagnosis, median (IQR) (years)	6·6 (4·0–10·3)	6·6 (4·0–10·3)
Time before seroconversion, median (IQR) (months)	5 (3–6)	n.a.
Time before diagnosis, median (IQR) (months)	53 (22–79)	6 (2–19)

T1D = type 1 diabetes; IQR = interquartile range; n.a. = not available.

### Tetramer assembly

Soluble, fluorochrome‐conjugated peptide‐HLA class I tetramers were generated as described previously 51. The peptide–human leucocyte antigen (pHLA)‐B*3906 tetramers were manufactured with PPI_5–12_ test peptides 43 and EBV BMRF1_268–276_ control peptides 43, 52. The pHLA‐A*2402 tetramers were manufactured with PPI_3–11_ and InsB_15–24_ test peptides 13, 53. Epitope sequences are given in Table 4. Tetramers were assembled over five separate 20‐min steps with the successive addition of streptavidin–allophycocyanin (APC) (Life Technologies, Carlsbad, CA, USA) to monomeric pHLA at a molar streptavidin : pHLA ratio of 1 : 4. Phosphate‐buffered saline (PBS) was added to give a final multimer concentration of 0·1 mg/ml pHLA content. Tetramers were stored in the dark at 4°C and used on the same day as assembly.

**Table 4:** HLA‐B*3906 and HLA‐A*2402‐restricted CD8 T cell epitopes

Epitope	HLA class I restriction	Sequence
PPI_3–11_	A*2402	LWMRLLPLL
InsB_15–24_	A*2402	LYLVCGERGF
PPI_5–12_	B*3906	MRLLPLLA
EBV BMRF1_268–276_	B*3906	YRSGIIAVV

PPI = preproinsulin; HLA = human leucocyte antigen; EBV = Epstein–Barr virus.

### Cell staining and flow cytometry

Thawed peripheral blood mononuclear cells (PBMC) were washed in RPMI supplemented with 2% human AB serum; 3–4 million PBMCs per stain were transferred to flow cytometry tubes, washed with PBS and stained with a live/dead aqua amine reactive dye (Invitrogen, Carlsbad, CA, USA) for 10 min at room temperature, followed by washing with PBS. PBMCs were incubated with the protein kinase inhibitor dasatinib (Axon Medchem, Reston, VA, USA) at a final concentration of 50 nM for 30 min at 37°C to enhance the detection of low‐avidity T cells 12, 54 and C‐C chemokine receptor type 7 (CCR7) BV421 monoclonal antibody (Biolegend, San Diego, CA, USA). Subsequently, PBMCs were stained for 10 min at 37°C with 1 μg of pHLA tetramer per tube. After washing with fluorescence activated cell sorter (FACS) buffer (PBS supplemented with 2% human AB serum and 3% fetal calf serum), PBMCs were stained with mouse anti‐APC unconjugated monoclonal antibody (Biolegend) at a concentration of 10 mg/ml on ice for 20 min to stabilize binding of APC‐labelled tetramers 55. After washing with FACS buffer, cells were stained with the following surface monoclonal antibodies/fluorophores for 20 min on ice: CD3 BV785, CD8 phycoerythrin cyanin 7 (PECy7), CD27 BV605 (all Biolegend); CD14 V500, CD16 V500, CD19 V500, CD45RA PE‐CF594, CD95 PE, CD57 fluorescein isothiocyanate (FITC) (all BD Biosciences, San Jose, CA, USA) and CD4 PECy5.5 (Invitrogen). Stained PBMCs were kept on ice and in the dark until acquisition on the same day on an LSR Fortessa (BD Biosciences). Gates to define tetramer positivity were set based on PBMC samples which were not stained with tetramer. No CD8 T cells were stained in the absence of tetramers. We elected to use this method to define tetramer‐positive cells as control tetramers incorporating irrelevant self‐peptides are likely to stain some T cells, while control tetramers incorporating foreign peptides from previously unencountered viruses have been found to stain some T cells in seronegative donors 56. Within the antigen‐specific CD8 T cell populations and total CD8 T cell populations, CD8 T cell subsets were assessed using the following cell surface marker combinations: naive (N;CCR7^+^CD45RA^+^CD27^+^CD57^–^CD95^–^), stem‐cell memory‐like (SCM; CCR7^+^CD45RA^+^CD27^+^CD57^–^CD95^+^), central memory (CM; CCR7^+^CD45RA^–^CD27^+^), transitional memory (TM; CCR7^–^CD45RA^–^CD27^+^), effector memory (EM; CCR7^–^CD45RA^–^CD27^–^), terminal effector (TE; CCR7^–^CD45RA^+^). The gating strategy is illustrated in Supporting information, Fig. S1. Flow cytometry data were analysed using FlowJo version 9.4 (Tree Star, Ashland, OR, USA). Due to limitations in cell numbers available of some samples, tetramer staining was limited as follows: two HLA‐B*3906 ^+^ control subjects were not stained with EBV tetramer; two HLA‐A*2402 ^+^ control subjects, six HLA‐A*2402 ^+^ prediagnosis subjects and five HLA‐A*2402 ^+^ pre‐seroconversion subjects were not stained with InsB tetramer.

### Dual‐colour tetramer staining

Dual‐colour staining was performed with the newly developed pHLA‐B*3906 tetramers to verify the specificity of tetramer‐binding cells (Supporting information, Fig. S2). The pHLA‐B*3906 tetramers manufactured with PPI_5–12_ and EBV BMRF1_268–276_ were assembled over five separate 20‐min steps with the successive addition of streptavidin–APC or streptavidin–PE (both Life Technologies) to monomeric pHLA at a molar streptavidin : pHLA ratio of 1 : 4. PBS was added to give a final multimer concentration of 0·1 mg/ml pHLA content. Thawed PBMC were stained as described above.

### Statistics

Comparisons between subject groups were performed using non‐parametric Mann–Whitney *U*‐tests. Paired comparisons between groups were performed using the non‐parametric Wilcoxon paired test. Statistical analysis was performed using GraphPad Prism version 7.0e.

## Results

### HLA‐B*3906‐restricted autoreactive CD8^+^ T cells in type 1 diabetes

**Figure 1:**
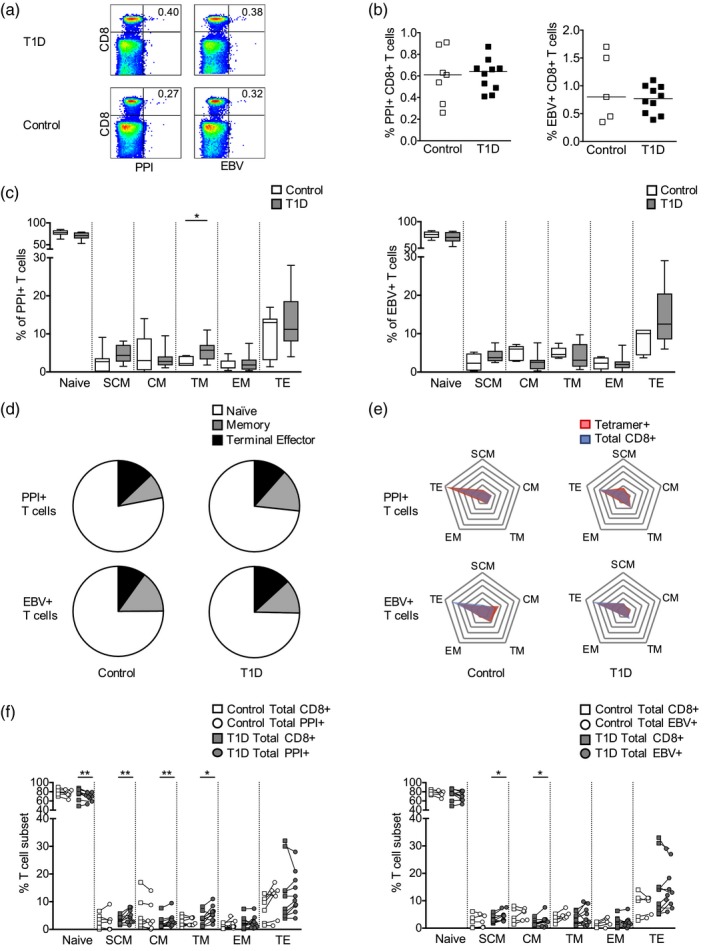
Frequency and phenotype of human leucocyte antigen (HLA)‐B*3906‐restricted preproinsulin (PPI)‐ and Epstein–Barr virus (EBV)‐specific CD8^+^ T cells in patients with newly diagnosed type 1 diabetes and control subjects. (a) Representative tetramer staining of peripheral blood mononuclear cells (PBMC) from type 1 diabetes (T1D) subjects (*n* = 10) and controls (*n* = 7). (b) Frequency of PPI_5–12_‐ and EBV BMRF1_268–276_‐specific CD8^+^ T cells. Mann–Whitney *U*‐tests *P* > 0·05. (c) Phenotype of PPI_5–12_‐ and EBV BMRF1_268–276_‐specific CD8^+^ T cells. Mann–Whitney *U*‐tests; *P < 0·05. (d) Median T cell subset percentages for PPI_5–12_‐ and EBV BMRF1_268–276_‐specific CD8^+^ T cells in T1D and controls. Memory: pooled stem cell‐like memory, central memory, transitional memory and effector memory. (e) Frequency of memory and effector T cell subsets expressed as a percentage of non‐naive T cells within tetramer‐specific (red) and polyclonal (blue) CD8^+^ T cell populations. Radial lines represent intervals of T cell subset frequencies of 10% from 0 to 60%, with the lowest value at the centre and the highest value at the periphery. Polygons link the frequency of each T cell subset. (f) Phenotype of PPI_5–12_‐ and EBV BMRF1_268–276_‐specific CD8^+^ T cells (circles) compared to polyclonal CD8^+^ T cells (squares). Wilcoxon paired tests; *P < 0·05, **P < 0·01. Abbreviations: SCM: stem cell‐like memory, CM: central memory; TM: transitional memory; EM: effector memory; TE: terminal effector.

As HLA‐B*39 carries the strongest type 1 diabetes risk of all HLA class I gene polymorphisms, we sought to determine whether HLA‐B*39‐restricted CD8^+^ T cells with specificity for β cell antigens could be detected in individuals with type 1 diabetes. In particular, we examined HLA‐B*3906‐restricted CD8^+^ T cells specific for the preproinsulin epitope PPI_5–12_ by staining *ex‐vivo* PBMC samples with pHLA class I tetramers. As a control, we analysed CD8^+^ T cells specific for the HLA‐B*3906‐restricted EBV lytic cycle protein epitope BMRF1_268–276_. We assessed the frequency and phenotype of antigen‐specific CD8^+^ T cells in PBMC samples obtained within 10 days of type 1 diabetes diagnosis from children aged up to 5 years and in non‐diabetic, age‐matched and HLA‐matched control subjects (Table 1). Two subjects with type 1 diabetes and one control subject tested positive for EBV antibodies (data from these subjects are highlighted in Supporting information, Fig. S3). Representative tetramer staining is shown in Fig. 1a. The frequency of PPI_5–12_‐specific and EBV BMRF1_268–276_‐specific CD8^+^ T cells was found to be similar in type 1 diabetes subjects and control subjects (Fig. 1b). Phenotypical analysis revealed that in subjects with type 1 diabetes there was a higher percentage of PPI_5–12_‐specific transitional memory CD8^+^ T cells compared to control subjects (Fig. 1c). In contrast, no significant differences were observed in the phenotype of EBV BMRF1_268–276_‐specific CD8^+^ T cells in subjects with type 1 diabetes compared to control subjects (Fig. 1c). The median percentages of naïve, memory and terminal effector cells comprising the PPI_5–12_‐ and EBV BMRF1_268–276_‐specific CD8^+^ T cell populations are represented in Fig. 1d. The pie charts illustrate the higher percentage of PPI_5–12_‐specific memory CD8^+^ T cells in subjects with type 1 diabetes compared to control subjects and the similarity in the percentages of EBV BMRF1_268–276_‐specific CD8^+^ T cell subsets in subjects with type 1 diabetes and control subjects (Fig. 1d).

In order to determine whether distinctive phenotypical characteristics were present in the antigen‐specific CD8^+^ T cells, we compared their phenotypes with those found in the polyclonal CD8^+^ T cell population within each PBMC sample. We used spider plots to visualize the difference in the memory and effector T cell subsets comprising the PPI_5–12_‐specific, EBV BMRF1_268–276_‐specific and polyclonal CD8^+^ T cell populations in subjects with type 1 diabetes and control subjects (Fig. 1e). For example, in subjects with type 1 diabetes, we observe a larger proportion of memory T cell subsets in the PPI_5–12_‐specific CD8^+^ T cell population compared to the polyclonal CD8^+^ T cell population, illustrated by a red polygon (representing PPI‐specific T cells) that is more expanded along the arcs of the web compared to the blue polygon (representing polyclonal CD8^+^ T cells) (Fig. 1e). Paired comparisons of tetramer‐specific and polyclonal CD8^+^ T cells revealed that in subjects with type 1 diabetes, the PPI_5–12_‐specific CD8^+^ T cell population was comprised of significantly fewer naive cells and significantly more stem‐cell‐like memory cells, central memory cells and transitional memory cells compared to the polyclonal CD8^+^ T cell population (Fig. 1f). Therefore, the PPI_5–12_‐specific CD8^+^ T cells display a more antigen‐experienced phenotype compared to polyclonal CD8^+^ T cells in type 1 diabetes subjects. Importantly, these differences were not observed within the PPI_5–12_‐specific CD8^+^ T cell population in control subjects, indicating that autoreactive T cells acquire this phenotype as a result of the disease process (Fig. 1f). In EBV‐specific CD8^+^ T cell populations, there was an increase in stem cell memory‐like and central memory cells compared to the polyclonal CD8^+^ T cell population in type 1 diabetes subjects, and we did not detect significant phenotypical differences compared to polyclonal CD8^+^ T cells in control subjects (Fig. 1f). The phenotype of polyclonal CD8^+^ T cells was not significantly different between type 1 diabetes subjects and control subjects (Supporting information, Fig. S4). A comparison of the data from the autoantibody‐positive and ‐negative control subjects is shown in Supporting information, Fig. S5.

Additionally, we had the opportunity to assess PPI_5–12_‐specific CD8^+^ T cells 5 years prior to the diagnosis of type 1 diabetes in an HLA‐B*3906 ^+^ child who was aged 3·3 years at the time of blood draw (Supporting information, Fig. S6a). In this individual, we observed a higher frequency of PPI_5–12_‐specific transitional memory CD8^+^ T cells compared to control subjects, as we observed in patients with type 1 diabetes (Supporting information, Fig. S6b). Compared to the polyclonal CD8^+^ T cell population, in the PPI_5–12_‐specific CD8^+ ^T cell population we observed fewer naive cells and more central memory cells, transitional memory cells, effector memory cells and terminal effector cells, indicative of an antigen‐experienced phenotype (Supporting information, Fig. S6c,d). Although we were not able to compare samples from this case before and after diagnosis, the phenotypical characteristics of PPI_5–12_‐specific CD8^+^ T cells in the subject sampled 5 years before diagnosis appear to be similar to the HLA‐B*3906 ^+^ subjects sampled at the time of diagnosis of type 1 diabetes.

### HLA‐A*2402‐restricted autoreactive CD8^+^ T cells in type 1 diabetes

**Figure 2:**
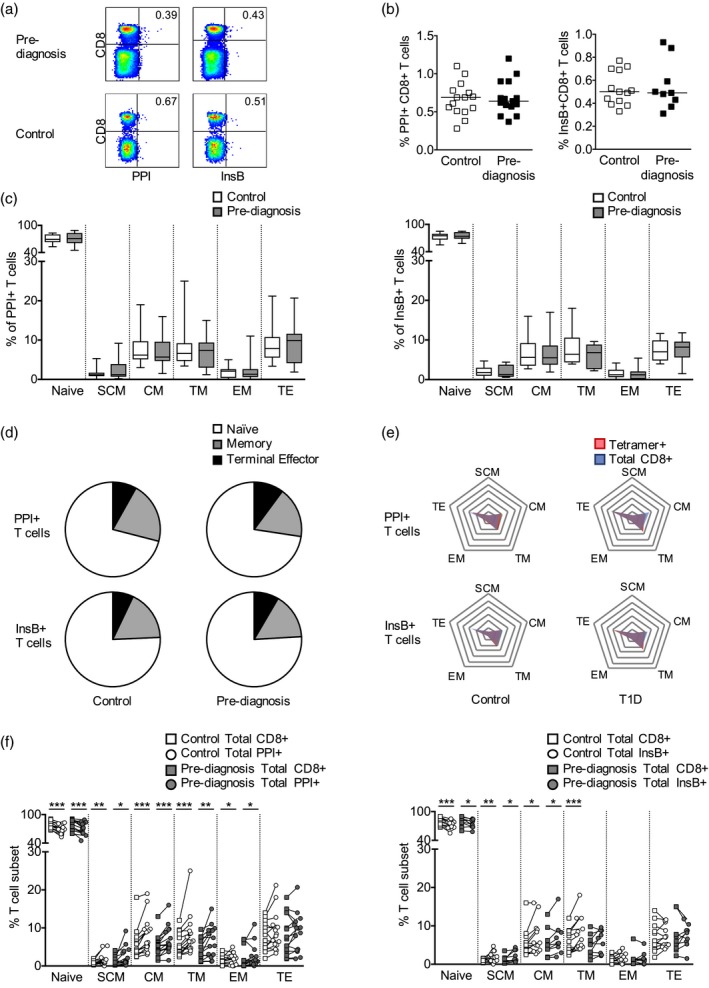
Frequency and phenotype of human leucocyte antigen (HLA)‐A*2402‐restricted preproinsulin (PPI)‐ and insulin B (InsB)‐specific CD8^+^ T cells before the diagnosis of type 1 diabetes (T1D) in affected subjects and control subjects. (a) Representative tetramer staining of peripheral blood mononuclear cells (PBMC) from subjects before T1D diagnosis (*n* = 17) and controls (*n* = 17). (b) Frequency of PPI_3–11_ and InsB_15–24‐_specific CD8^+^ T cells. Mann–Whitney *U*‐tests *P* > 0·05. (c) Phenotype of PPI_3–11_ and InsB_15–24_‐specific CD8^+^ T cells. (d) Median T cell subset percentages for PPI_3–11_ and InsB_15–24‐_specific CD8^+^ T cells. Memory: pooled stem cell‐like memory, central memory, transitional memory and effector memory. (e) Frequency of memory and effector T cell subsets expressed as a percentage of non‐naive T cells within tetramer‐specific (red) and polyclonal (blue) CD8^+^ T cell populations. Radial lines represent intervals of T cell subset frequencies of 5% from 0 to 35%, with the lowest value at the centre and the highest value at the periphery. Polygons link the frequency of each T cell subset. (f) Phenotype of tetramer‐specific CD8^+^ T cells (squares) compared to polyclonal CD8^+^ T cells (circle). Wilcoxon paired tests *P < 0·05, **P < 0·01, ***P < 0·001. Abbreviations: SCM: stem cell‐like memory, CM: central memory; TM: transitional memory; EM: effector memory; TE: terminal effector.

Using a similar approach, we studied CD8^+^ T cells recognizing β cell peptides presented by HLA‐A*2402 in children followed from birth to clinical diagnosis. PPI_3–11_‐ and InsB_15–24_‐specific pHLA tetramers were used to stain PBMCs from 15 children ascertained before clinical diagnosis and 15 age‐, sex‐ and HLA‐matched control subjects (Fig. 2a). We found that the frequencies of circulating PPI_3–11_‐ and InsB_15–24_‐specific CD8^+^ T cells were similar in subjects with type 1 diabetes and control subjects (Fig. 2b), and the subject groups did not differ significantly in terms of the phenotype of PPI_3–11_‐ or InsB_15–‐24_‐specific CD8^+^ T cells (Fig. 2c,d). However, when antigen‐specific and polyclonal CD8^+^ T cell populations were compared, PPI_3–11_‐ and InsB_15–24_‐specific CD8^+^ T cell populations in type 1 diabetes and control subjects contained significantly fewer naive cells and significantly more stem cell‐like memory, central memory and transitional memory cells compared to polyclonal CD8^+^ T cell populations (Fig. 2e,f). In addition, PPI_3–11_‐specific T cell populations also contained significantly more effector memory cells than polyclonal CD8^+^ T cell populations (Fig. 2f). The phenotype of polyclonal CD8^+^ T cells was not significantly different between type 1 diabetes subjects and control subjects (Supporting information, Fig. S7). Therefore, PPI_3–11_‐ and InsB_15–24_‐specific CD8^+^ T cells in both subject groups showed a more antigen‐experienced phenotype compared to polyclonal CD8^+^ T cells.

**Figure 3:**
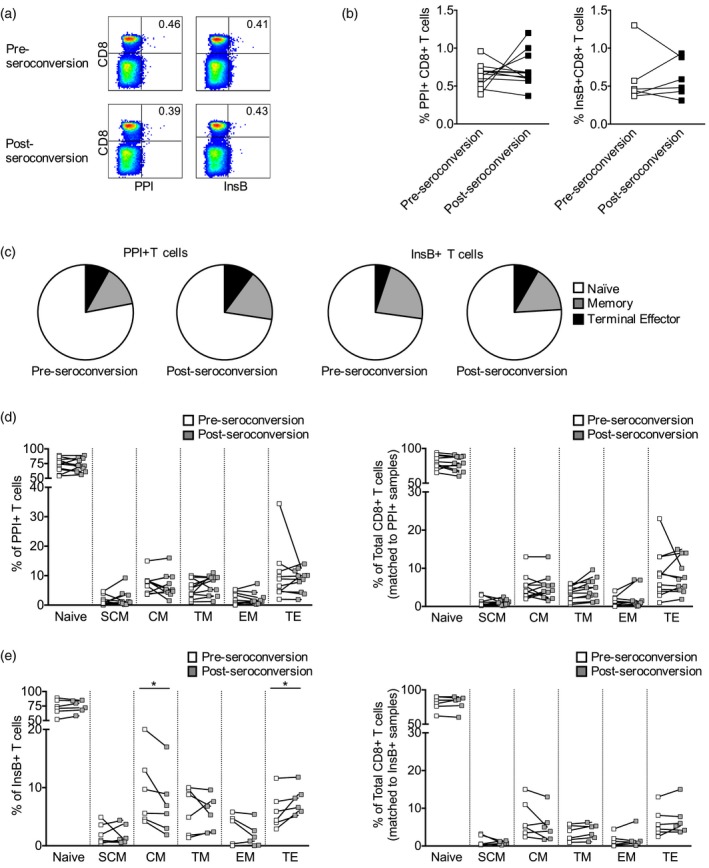
Frequency and phenotype of human leucocyte antigen (HLA)‐A*2402‐restricted preproinsulin (PPI)_3–11_‐ and InsB_15–24_‐specific CD8^+^ T cells before and after seroconversion to autoantibody positivity. (a) Representative tetramer staining of longitudinal peripheral blood mononuclear cells (PBMC) samples (*n* = 11). (b) Frequency of PPI_3–11_ and InsB_15–24_‐specific CD8^+^ T cells. Wilcoxon paired tests were used to compare groups (*P* > 0·05). (c) Median T cell subset percentages for PPI_3–11_ and InsB_15–24_‐specific CD8^+^ T cells. Memory: pooled stem cell‐like memory, central memory, transitional memory and effector memory. (d) Phenotype of PPI_3–11_‐specific and polyclonal CD8^+^ T cells. Wilcoxon paired tests were used for comparisons (*P* > 0·05). (e) Phenotype of InsB_15–24_‐specific and polyclonal CD8^+^ T cells. Wilcoxon paired tests were used for comparisons; *P < 0·05. Abbreviations: SCM: stem cell‐like memory, CM: central memory; TM: transitional memory; EM: effector memory; TE: terminal effector.

In order to evaluate changes in autoreactive HLA‐A*2402‐restricted CD8^+^ T cell populations before and after the initiation of islet autoimmunity, we analysed the frequency and phenotype of β cell‐specific CD8^+^ T cells in longitudinal samples from 11 HLA‐A*2402 ^+^ children ascertained before seroconversion to autoantibody positivity (median = 5 months) compared to samples taken before clinical diagnosis after the appearance of autoantibodies (median = 2 months before diagnosis) (Table 4, Fig. 3a). The frequencies of PPI_3–11_‐ and InsB_15–24_‐specific CD8^+^ T cells were not found to be significantly different in the samples taken before and after seroconversion (Fig. 3b). When we compared the phenotype of PPI_3–11_‐specific CD8^+^ T cells before seroconversion and before diagnosis, we found it was similar (Fig. 3c,d). However, within the InsB_15–24_‐specific CD8^+^ T cell population, we observed that the percentage of terminal effector cells was increased and the percentage of central memory cells was decreased in samples taken after seroconversion relative to samples taken before seroconversion (Fig. 3c,e). In contrast, the phenotype of the polyclonal CD8^+^ T cell population remained similar in pre‐ and post‐seroconversion samples (Fig. 3e). Collectively, these results provide evidence that β cell‐reactive CD8^+^ T cells restricted by disease‐associated HLA class I molecules display an antigen‐experienced phenotype and acquire enhanced effector function during the period leading to clinical diagnosis.

## Discussion

The HLA class I HLA‐B*39 allele confers the strongest risk of type 1 diabetes of all HLA class I gene polymorphisms 17. This is the first study to report autoreactive CD8^+^ T cells restricted by HLA‐B*3906 in blood samples from individuals with type 1 diabetes. Here we show that the transitional memory subset of B*3906‐restricted PPI‐specific transitional memory CD8^+^ T cells comprises a higher proportion of the total PPI‐specific population in HLA‐B*3906 ^+^ children with new‐onset type 1 diabetes compared to HLA‐matched control subjects. Transitional memory cells have a differentiation status that is intermediate between that of central memory cells and effector memory cells in terms of phenotype, transcription factor expression and cytokine‐induced proliferation 57, 58, 59 **.** According to the recently proposed model of progressive differentiation, a naive T cell will differentiate gradually to different memory subsets through stem cell‐like memory, central memory, transitional memory, effector memory and terminal effector, progressively losing or acquiring specific functions 60. Central memory cells home to secondary lymph nodes and are better equipped to persist and proliferate in response to antigen, whereas effector memory cells are more cytolytic and express integrins and chemokine receptors that facilitate their entry into inflamed tissues 61. So far, a distinct pathophysiological role for the intermediate transitional memory subset has not been described. Here we defined transitional memory cells as having a cell surface expression marker profile of CCR7^–^CD45RA^–^CD27^+^, where the absence of CCR7 expression suggests that these cells have been activated in lymph nodes and are capable of homing to inflamed islets. Studies in non‐human primates have reported that transitional memory CD8^+^ T cells have higher expression levels of the transcription factors eomesodermin (EOMES), aryl hydrocarbon receptor (AHR) and RAR‐related orphan receptor C (RORC) compared to central memory and effector memory cells 57. The major role of EOMES is to maintain a memory CD8^+^ T cell repertoire capable of expansion on re‐encountering antigen. EOMES also functions as a master regulator of cell‐mediated immunity that controls genes encoding effector molecules including IFN‐γ, granzyme B and perforin 62, 63, 64. This would indicate that, like effector memory cells, transitional memory cells are able to execute target cell killing. As far as we know, a role for antigen‐specific transitional memory CD8^+^ T cells in autoimmune pathology has not yet been described. However, the capability of transitional memory cells to home to tissues and mediate cytotoxic effector functions suggests that the higher frequency of PPI_5–12_‐specific transitional memory cells we have detected in patients affected by type 1 diabetes has the potential to directly mediate β cell killing.

Further observations were made by pairwise comparison of functional phenotypes of HLA‐B*3906‐restricted PPI_5–12_‐specific CD8^+^ T cell populations with those of polyclonal CD8^+^ T cells, the latter representing a synthesis of all CD8^+^ T cell‐mediated antigen experiences in each subject. The aim of these analyses was to identify functional phenotypes specifically expanded by autoantigens. We noted that multiple memory T cell subsets were enriched within PPI_5–12_‐specific CD8^+^ T cell populations in multiple memory T cell subsets in children with type 1 diabetes, but this was not observed in non‐diabetic control subjects. An increase in PPI_5–12_‐specific stem cell‐like memory cells, central memory cells and transitional memory cells was accompanied by a reduction in naive cells compared to the polyclonal CD8^+^ T cell population. Memory CD8^+^ T cells are poised for rapid proliferative responses, and to execute cytotoxic functions and secrete effector cytokines upon re‐encountering their cognate antigen 61, 65, 66, 67. Therefore, the enrichment of β cell‐specific memory CD8^+^ T cell populations at the time of clinical diagnosis indicates that newly diagnosed type 1 diabetes is characterized by antigen‐driven differentiation of HLA‐B*3906‐restricted CD8^+^ T cells, probably facilitated by a tissue‐specific inflammatory process. Collectively, our findings suggest that enrichment of PPI_5–12_‐specific memory CD8^+^ T cell subsets in subjects with type 1 diabetes compared to non‐diabetic control subjects and polyclonal CD8^+^ T cells may reflect a driver role in relation to β cell killing, consistent with the accelerated loss of β cells that has been observed in HLA‐B*3906 ^+^ individuals, relative to patients with other HLA class I alleles 17, 20, 21, 22, 23, 24. HLA‐B*3906 is also known to associate with a relatively younger age of diagnosis and notably, this allele has been reported to be more frequent in children who progress to clinical type 1 diabetes under the age of 5·5 years 20, an age group which corresponds with the cohort examined in this study.

Clinical diagnosis of type 1 diabetes is an event marking the late stages of the destructive autoimmune process, when the number of functional β cells falls below the threshold required for adequate insulin production. However, the first signs of autoimmunity emerge months or years earlier with the appearance of several islet‐specific autoantibodies. In longitudinal samples from HLA‐A*2402 ^+^ children with high‐risk HLA class II genotypes, we found that CD8^+^ T cells specific for InsB_15–24_ had reduced frequencies of central memory cells and elevated frequencies of terminal effectors before diagnosis, relative to early‐stage disease before the appearance of autoantibodies. The percentage of terminal effector cells within total CD8^+^ T cell populations was within the range previously reported for children of a similar age 68, 69, 70, 71. Surprisingly, we observed phenotypical changes in CD8^+^ T cells with specificity for InsB_15–24_ but not PPI_3–11._ We speculate that InsB presentation may be enhanced by autoantibody‐mediated antigen presentation which will increase after seroconversion, whereas PPI, not being a part of mature insulin/proinsulin, may be targeted later by the autoimmune response via epitope spreading. While the concept of epitope spreading of autoantibody responses in preclinical type 1 diabetes has been well established 72, 73, 74, 75, 76, 77, intermolecular spreading of T cell responses has also been described during the preclinical period of type 1 diabetes 78, 79 and in animal models of diabetes 80, 81, 82, 83.

It is important to note that as none of the subjects examined in this study had received exogenous insulin, the changes we observed in InsB‐specific CD8^+^ T cells reflect the natural history of disease. Our results suggest that the natural evolution of the β cell‐specific CD8^+^ T cell response during the prediabetic period in young children becomes increasingly dominated by end‐stage cytolytic effector cells which are capable of directly mediating β cell destruction. These data fit with observations that at clinical diagnosis β cell destruction is already well advanced 84. Of interest, previous studies of the DIPP cohort have reported that the HLA‐A*24 allele is associated with accelerated disease progression from seroconversion to clinical disease 26. One interpretation is that HLA‐A*24‐restricted CD8^+^ T cells are potent mediators of β cell death after autoimmunity has been established. Furthermore, the presence of the HLA‐A*24 allele has also been associated with a reduced frequency of particular autoantibodies at clinical diagnosis 85, 86, 87. To explain this effect, it has been suggested that compared to other allotypes, HLA‐A*24‐restricted CD8^+^ T cells may mediate a more complete β cell destruction, resulting in a loss of antigenic stimulus, or alternatively, HLA‐A*24‐restricted CD8^+^ T cells may mediate a more rapid β cell destruction, thus attenuating inter‐ and intramolecular spreading of the autoimmune response 85, 86, 87. Our previous work showed that HLA‐A*2402‐restricted PPI‐specific CD8^+^ T cell clones derived from patients with type 1 diabetes are able to kill isolated β cells *in vitro*, exemplifying the potential for such cells to directly mediate β cell death in individuals with type 1 diabetes 88.

In this study, we observed that β cell‐specific CD8^+^ T cell frequencies in peripheral blood were similar in subjects with type 1 diabetes and control subjects. Similarly, other studies have found no significant differences between type 1 diabetes subjects and healthy donors in the frequencies of circulating CD8^+^ T cells reactive to multiple HLA‐A2‐restricted β cell epitopes 12, 56, 89. However, some studies have reported higher frequencies of β cell‐reactive CD8^+^ T cells in HLA‐A*02 ^+^ or HLA‐A*24 ^+^ type 1 diabetes subjects compared to healthy donors 13, 89, 90, 91, and it is important to take into account differences in methods (such as dasatinib enhancement of tetramer staining) and study populations (i.e. adults *versus* children).

In summary, we have demonstrated that cytotoxic T cells restricted by disease‐associated HLA class I molecules display an antigen‐experienced phenotype and acquire enhanced effector function during the period leading to clinical diagnosis, suggesting that these cells have a critical role in driving β cell destruction and may provide useful biomarkers of disease activity in monitoring progression to type 1 diabetes.

## Disclosures

The authors have declared that no conflicts of interest exist.

## Author contributions

L. Y., I. P. A. and R. B. performed the experiments, evaluated data and applied statistical analysis. G. D., K. T., S. H. and A. K. S. contributed to tetramer assembly and staining and flow cytometry experiments. R. V., J. T. and M. K. were responsible for recruitment and follow‐up of children in the DIPP project. R. V. and J. T. also participated in the Finnish Pediatric Diabetes Register as investigators. T. H. and M. K. were responsible for recruitment and follow‐up of children in in the Finnish Pediatric Diabetes Register. M. K. is the principal investigator of the Finnish Pediatric Diabetes Register. J. I., T. H. and M. K. provided clinical samples and M.‐L. M. and J. I. provided HLA genotyping data. L. Y. wrote the draft. M. P. and J. I. designed the study. All authors had the opportunity to discuss the results and comment on the manuscript.

## Supporting information


**Fig. S1.** **Flow cytometry gating strategy for determination of antigen‐specific CD8 T cell subsets**. **(a)** Lymphocytes were defined as CD3 positive, ‘Dump’ (dead cell stain, CD14, CD16, CD19) negative gated on FSC and SSC characteristics. Doublets and CD4^+^CD8^+^ double‐positive cells were excluded. Cells were gated as CD8^+^Tetramer^+^ or CD8^+^Tetramer‐. **(b)** Gating of CD8^+^ T cell subsets: Naïve (N;CCR7^+^CD45RA^+^CD27^+^CD57‐CD95‐), stem cell memory‐like (SCM; CCR7^+^CD45RA^+^CD27^+^CD57‐CD95^+^), central memory (CM; CCR7^+^CD45RA‐CD27^+^), transitional memory (TM; CCR7‐CD45RA‐CD27^+^), effector memory (EM; CCR7‐CD45RA‐CD27‐) and terminal effector (TE; CCR7‐CD45RA^+^). **(c)** Schematic of the CD8^+^ T cell subsets analysed and the cell surface markers used for their definition.

**Fig. S2.** Dual‐colour tetramer staining with pHLA‐B*3906 tetramers loaded with PPI_5‐12_ and EBV BMRF1_268‐276._ (a) Gating strategy used to identify double‐positive tetramer‐binding CD8^+^ T cells. Lymphocytes were defined as CD3^+^, ‘Dump’ (dead cell stain, CD14, CD16, CD19)‐negative, and gated on lymphocyte SSC and FSC characteristics. Doublets were excluded. **(b)** PBMC from an HLA‐B*3906 ^+^ donor stained with PPI_5‐12_ and EBV BMRF1_268‐276_ pHLA‐B*3906 tetramers which were dual‐labelled with APC and PE. The majority of tetramer‐binding cells are double‐positive for both tetramers.

**Fig. S3.** Frequency and phenotype of HLA‐B*3906‐restricted EBV‐specific CD8^+^ T cells in EBV antibody‐positive (*n* = 3) and antibody‐negative (*n* = 9) subjects. (a) Frequency of EBV BMRF1_268‐276_‐specific CD8^+^ T cells in EBV antibody‐positive (red) and antibody‐negative (white) subjects. **(b)** Phenotype of EBV BMRF1_268‐276_‐specific CD8^+^ T cells in EBV antibody‐positive (red) and antibody‐negative (white) subjects.

**Fig. S4.** Phenotype of polyclonal CD8^+^ T cell populations in HLA‐B*3906^+^ newly diagnosed type 1 diabetes subjects and HLA‐B*3906^+^ control subjects. The phenotype of polyclonal CD8^+^ T cells was not found to be significantly different between HLA‐B*3906^+^ T1D subjects (grey) and healthy controls (white). Mann–Whitney U‐tests *P* > 0·05.

**Fig. S5.** Frequency and phenotype of HLA‐B*3906‐restricted PPI‐specific CD8^+^ T cells in autoantibody positive control (*n* = 1) and negative controls (*n* = 6). (a) Frequency and phenotype of PPI_5‐12_‐specific CD8^+^ T cells and polyclonal CD8^+^ T cells from autoantibody‐negative controls (white), autoantibody‐positive control (red) and T1D subjects (black). **(b)** Frequency and phenotype of EBV BMRF1_268‐276_‐specific CD8^+^ T cells and polyclonal CD8^+^ T cells from autoantibody‐negative controls (white), autoantibody‐positive control (red) and T1D subjects (black).

**Fig. S6.** Frequency and phenotype of HLA‐B*3906‐restricted PPI‐specific CD8^+^ T cells before the diagnosis of type 1 diabetes. (a) Frequency of PPI_5‐12_‐specific CD8^+^ T cells from an HLA‐B*3906 ^+^ type 1 diabetes subject before diagnosis (black) compared to HLA‐B*3906 ^+^ newly diagnosed type 1 diabetes subjects (grey) and HLA‐B*3906 ^+^ control subjects (white). **(b)** Phenotype of PPI_3‐11_‐specific CD8^+^ T cells from an HLA‐B*3906 ^+^ type 1 diabetes subject before diagnosis (black) compared to HLA‐B*3906 ^+^ newly diagnosed type 1 diabetes subjects (grey) and HLA‐B*3906 ^+^ control subjects (white). **(c)** Frequency of memory and effector T cell subsets expressed as a percentage of non‐naïve T cells within tetramer‐specific (red) and polyclonal (blue) CD8^+^ T cell populations. Radial lines represent intervals of T cell subset frequencies of 10% from 0 to 50%, with the lowest value at the centre and the highest value at the periphery. Polygons link the frequency of each T cell subset. **(d)** Phenotype of PPI_5‐12_‐specific CD8^+^ T cells (squares) compared to total polyclonal CD8^+^ T cells (circles) in an HLA‐B*3906 ^+^ type 1 diabetes subject before diagnosis (black), HLA‐B*3906 ^+^ newly diagnosed type 1 diabetes subjects (grey) and HLA‐B*3906 ^+^ control subjects (white).

**Fig. S7.** Phenotype of polyclonal CD8^+^ T cell populations in HLA‐A*2402^+^ type 1 diabetes subjects before diagnosis and HLA‐A*2402^+^ control subjects. The phenotype of polyclonal CD8^+^ T cells was not found to be significantly different between HLA‐A*2402^+^ pre‐diagnosis subjects (grey) and healthy controls (white). Mann–Whitney U‐tests *P* > 0.05.
