# Supplementary figures and images for: Circulating β cell‐specific CD8+ T cells restricted by high‐risk HLA class I molecules show antigen experience in children with and at risk of type 1 diabetes
Source: Clin Exp Immunol. 2019 Nov 10;199(3):263–77. doi: 10.1111/cei.13391 (PMC7008222; doi:10.1111/cei.13391)

Supplementary Figure 1

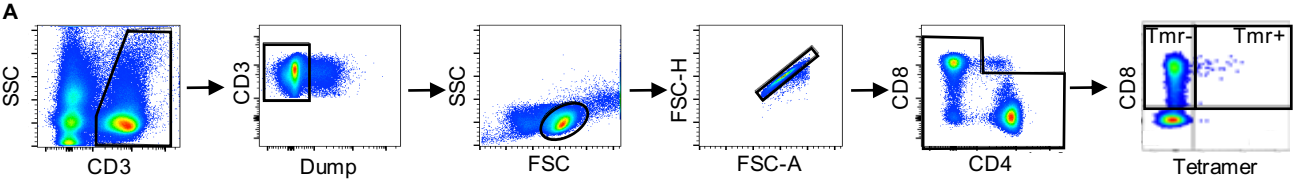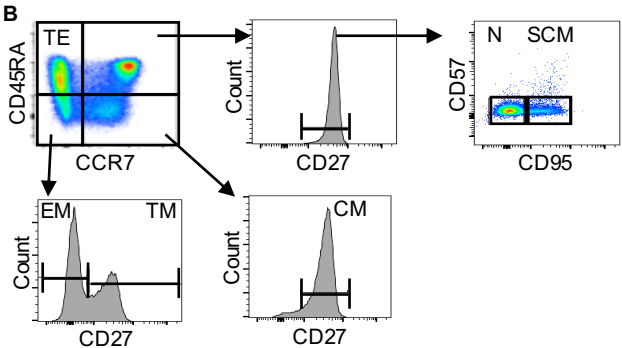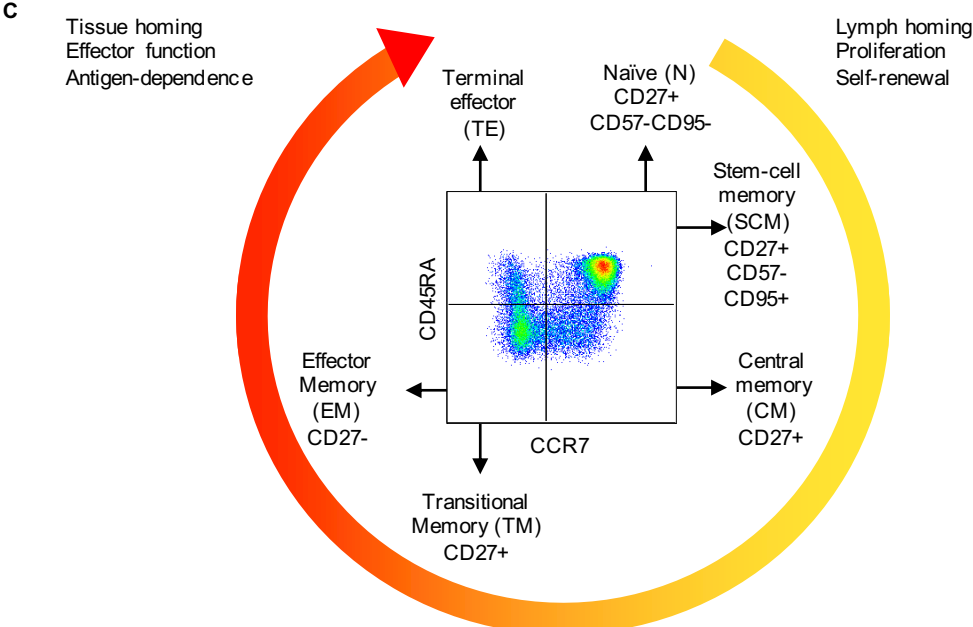

Supplement: Supplementary file 1 — Fig. S1. Flow cytometry gating strategy for determination of antigen‐specific CD8 T cell subsets. (a) Lymphocytes were defined as CD3 positive, ‘Dump’ (dead cell stain, CD14, CD16, CD19) negative gated on FSC and SSC characteristics. Doublets and CD4+CD8+ double‐positive cells were excluded. Cells were gated as CD8+Tetramer+ or CD8+Tetramer‐. (b) Gating of CD8+ T cell subsets: Naïve (N;CCR7+CD45RA+CD27+CD57‐CD95‐), stem cell memory‐like (SCM; CCR7+CD45RA+CD27+CD57‐CD95+), central memory (CM; CCR7+CD45RA‐CD27+), transitional memory (TM; CCR7‐CD45RA‐CD27+), effector memory (EM; CCR7‐CD45RA‐CD27‐) and terminal effector (TE; CCR7‐CD45RA+). (c) Schematic of the CD8+ T cell subsets analysed and the cell surface markers used for their definition. [file CEI-199-263-s001.pdf]

## Supplementary Figure 2

**A**

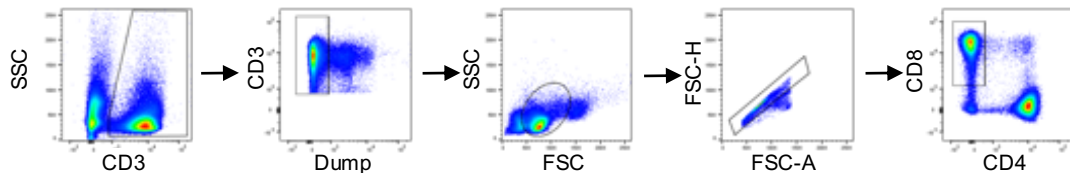

**B**

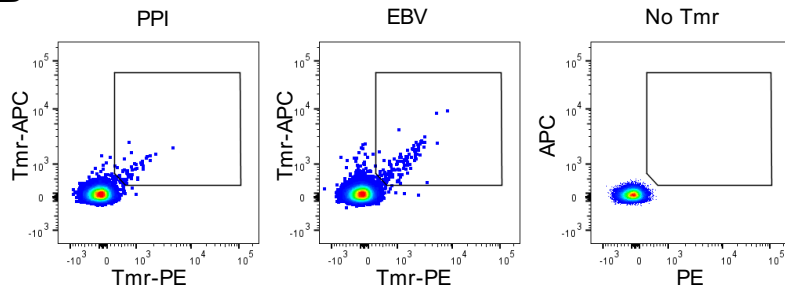

Supplement: Supplementary file 2 — Fig. S2. Dual‐colour tetramer staining with pHLA‐B*3906 tetramers loaded with PPI5‐12 and EBV BMRF1268‐276. (a) Gating strategy used to identify double‐positive tetramer‐binding CD8+ T cells. Lymphocytes were defined as CD3+, ‘Dump’ (dead cell stain, CD14, CD16, CD19)‐negative, and gated on lymphocyte SSC and FSC characteristics. Doublets were excluded. (b) PBMC from an HLA‐B*3906 + donor stained with PPI5‐12 and EBV BMRF1268‐276 pHLA‐B*3906 tetramers which were dual‐labelled with APC and PE. The majority of tetramer‐binding cells are double‐positive for both tetramers. [file CEI-199-263-s002.pdf]

# Supplementary Figure 3

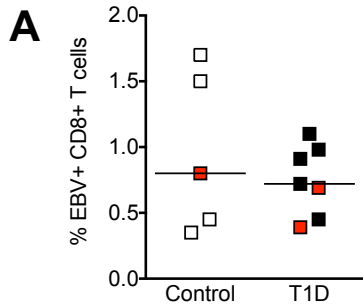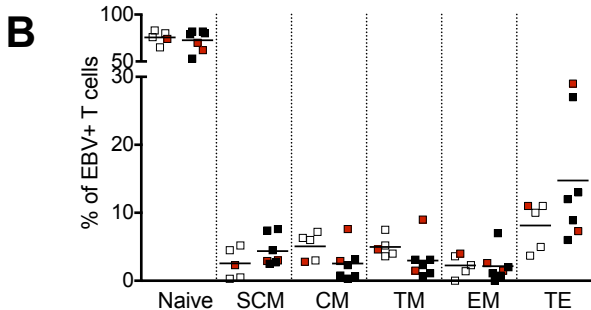

Supplement: Supplementary file 3 — Fig. S3. Frequency and phenotype of HLA‐B*3906‐restricted EBV‐specific CD8+ T cells in EBV antibody‐positive (n = 3) and antibody‐negative (n = 9) subjects. (a) Frequency of EBV BMRF1268‐276‐specific CD8+ T cells in EBV antibody‐positive (red) and antibody‐negative (white) subjects. (b) Phenotype of EBV BMRF1268‐276‐specific CD8+ T cells in EBV antibody‐positive (red) and antibody‐negative (white) subjects. [file CEI-199-263-s003.pdf]

# Supplementary Figure 4

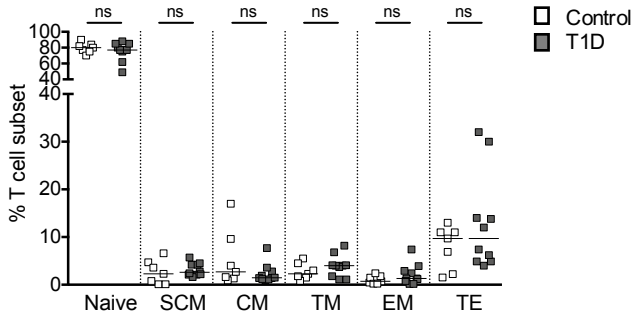

Supplement: Supplementary file 4 — Fig. S4. Phenotype of polyclonal CD8+ T cell populations in HLA‐B*3906+ newly diagnosed type 1 diabetes subjects and HLA‐B*3906+ control subjects. The phenotype of polyclonal CD8+ T cells was not found to be significantly different between HLA‐B*3906+ T1D subjects (grey) and healthy controls (white). Mann–Whitney U‐tests P > 0·05. [file CEI-199-263-s004.pdf]

Supplementary Figure 5

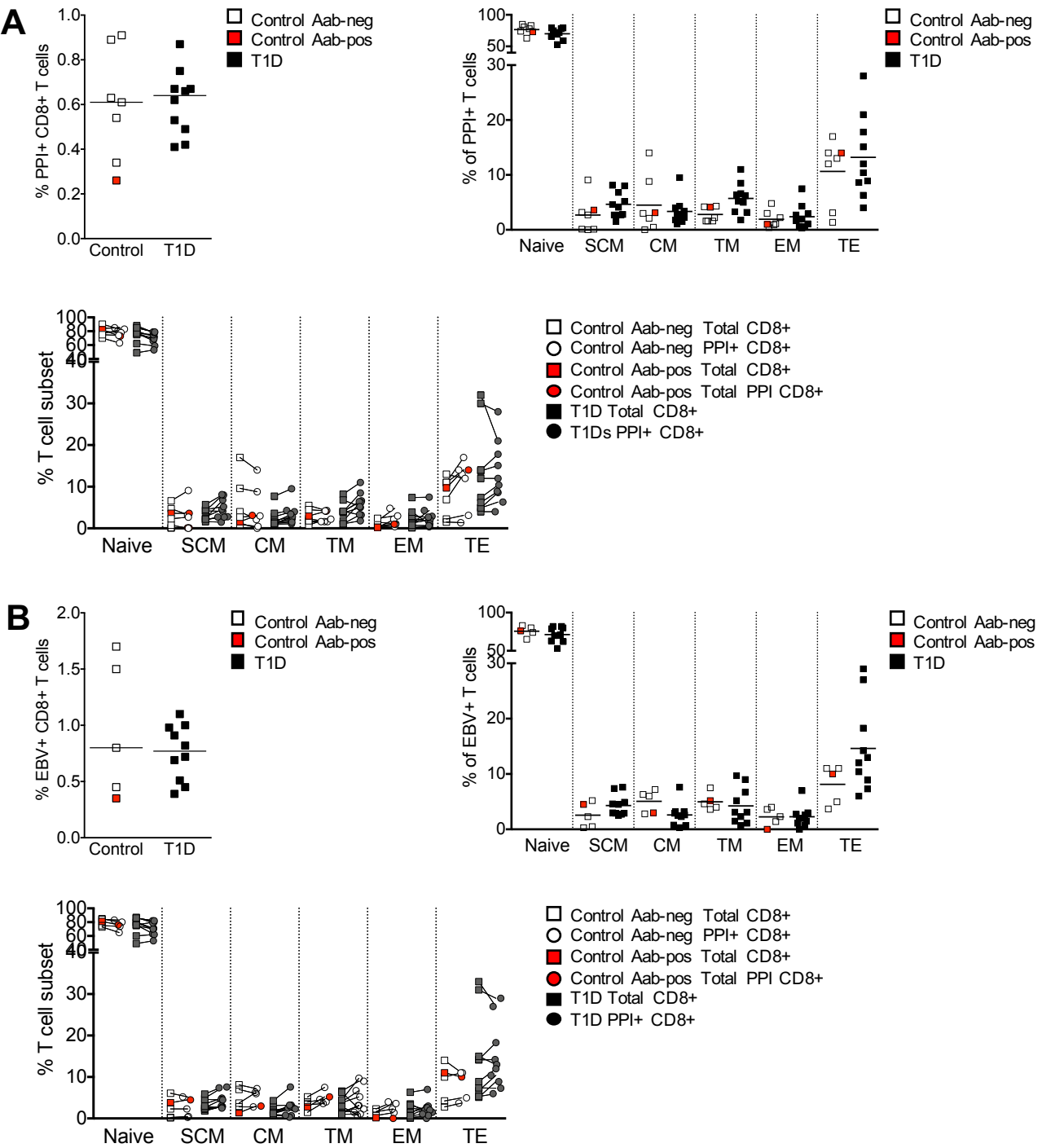

Supplement: Supplementary file 5 — Fig. S5. Frequency and phenotype of HLA‐B*3906‐restricted PPI‐specific CD8+ T cells in autoantibody positive control (n = 1) and negative controls (n = 6). (a) Frequency and phenotype of PPI5‐12‐specific CD8+ T cells and polyclonal CD8+ T cells from autoantibody‐negative controls (white), autoantibody‐positive control (red) and T1D subjects (black). (b) Frequency and phenotype of EBV BMRF1268‐276‐specific CD8+ T cells and polyclonal CD8+ T cells from autoantibody‐negative controls (white), autoantibody‐positive control (red) and T1D subjects (black). [file CEI-199-263-s005.pdf]

# Supplementary Figure 6

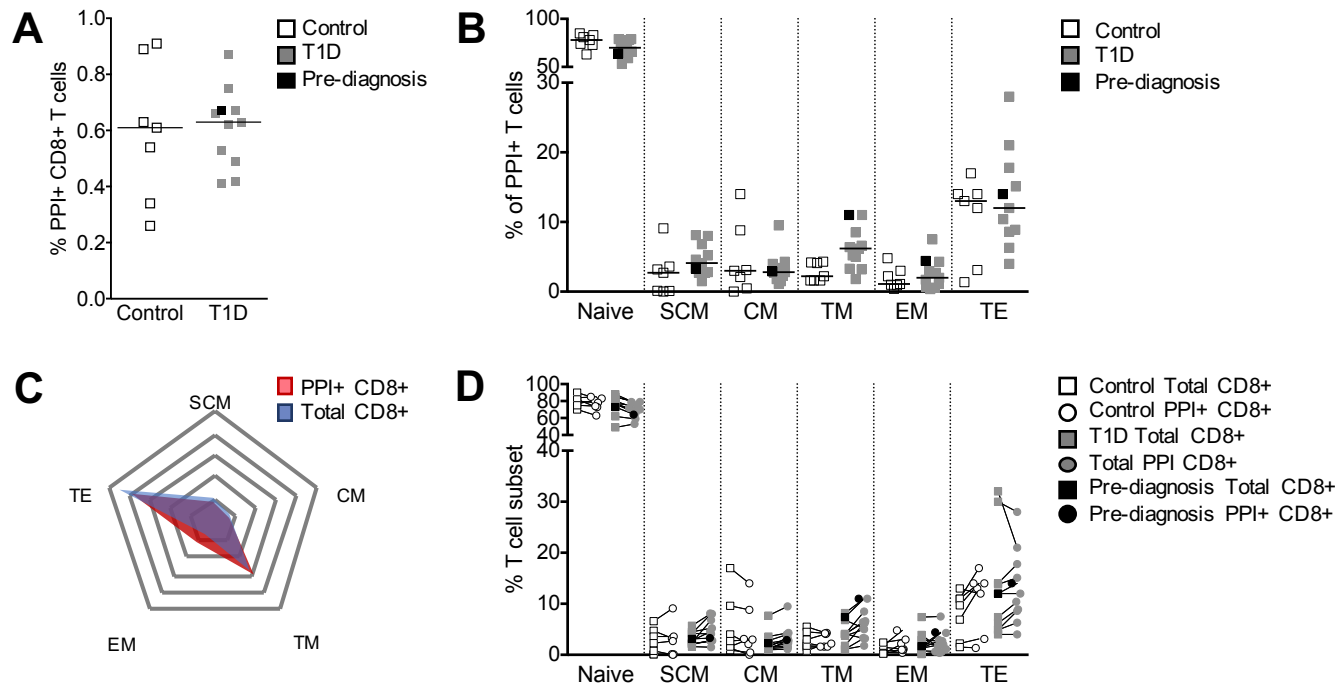

Supplement: Supplementary file 6 — Fig. S6. Frequency and phenotype of HLA‐B*3906‐restricted PPI‐specific CD8+ T cells before the diagnosis of type 1 diabetes. (a) Frequency of PPI5‐12‐specific CD8+ T cells from an HLA‐B*3906 + type 1 diabetes subject before diagnosis (black) compared to HLA‐B*3906 + newly diagnosed type 1 diabetes subjects (grey) and HLA‐B*3906 + control subjects (white). (b) Phenotype of PPI3‐11‐specific CD8+ T cells from an HLA‐B*3906 + type 1 diabetes subject before diagnosis (black) compared to HLA‐B*3906 + newly diagnosed type 1 diabetes subjects (grey) and HLA‐B*3906 + control subjects (white). (c) Frequency of memory and effector T cell subsets expressed as a percentage of non‐naïve T cells within tetramer‐specific (red) and polyclonal (blue) CD8+ T cell populations. Radial lines represent intervals of T cell subset frequencies of 10% from 0 to 50%, with the lowest value at the centre and the highest value at the periphery. Polygons link the frequency of each T cell subset. (d) Phenotype of PPI5‐12‐specific CD8+ T cells (squares) compared to total polyclonal CD8+ T cells (circles) in an HLA‐B*3906 + type 1 diabetes subject before diagnosis (black), HLA‐B*3906 + newly diagnosed type 1 diabetes subjects (grey) and HLA‐B*3906 + control subjects (white). [file CEI-199-263-s006.pdf]

# Supplementary Figure 7

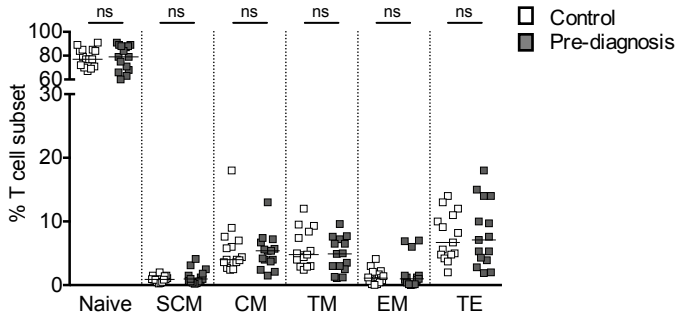

Supplement: Supplementary file 7 — Fig. S7. Phenotype of polyclonal CD8+ T cell populations in HLA‐A*2402+ type 1 diabetes subjects before diagnosis and HLA‐A*2402+ control subjects. The phenotype of polyclonal CD8+ T cells was not found to be significantly different between HLA‐A*2402+ pre‐diagnosis subjects (grey) and healthy controls (white). Mann–Whitney U‐tests P > 0.05. [file CEI-199-263-s007.pdf]
